# Data collection within patient support programs in Canada and implications for real-world evidence generation: the authors’ perspective

**DOI:** 10.3389/jpps.2023.11877

**Published:** 2023-10-13

**Authors:** Allison Wills, Arif Mitha, Winson Y. Cheung

**Affiliations:** ^1^ 20Sense Corp., Toronto, ON, Canada; ^2^ Department of Oncology, University of Calgary, Calgary, AB, Canada

**Keywords:** patient support programs, real world data, real world evidence, data collection, novel medications

## Abstract

Patient support programs (PSPs) offer a unique opportunity to collect real-world data that can contribute to improving patient care and informing healthcare decision making. In this perspective article, we explore the collection of data through PSPs in Canada, current advances in data collection methods, and the potential for generating acceptable real-world evidence (RWE). With PSP infrastructure already in place for most specialized drugs in Canada, adding and strengthening data collection capacities has been a focus in recent years. However, limitations in PSP data, including challenges related to quality, bias, and trust, need to be acknowledged and addressed. Forward-thinking PSP developers have been taking steps to strengthen the PSP datasphere, such as engaging third parties for data analysis, publishing peer-reviewed studies that utilize PSPs as a data source and incorporating quality controls into data collection processes. This article illustrates the current state of PSP data collection by examining six PSP RWE studies and outlining their data characteristics and the health outcomes collected from the PSP. A framework for collecting real-world data within a PSP and a checklist to address issues of trust and bias in PSP data collection is also provided. Collaboration between drug manufacturers, PSP vendors, and data specialists will be crucial in elevating PSP data to a level acceptable to healthcare decision makers, including health technology assessors and payers, with the ultimate beneficiary being patients.

## Introduction

Patient support programs (PSPs) exist to help patients navigate the clinical and logistical challenges of treatment with specialty medicines, which generally are high-cost, require specialized services, and treat chronic, serious, and rare diseases such as severe rheumatoid arthritis, multiple sclerosis (MS) and cancer [1–5]. Funded by drug manufacturers, PSPs address critical patient care gaps in the Canadian health care system, such as navigation of drug reimbursement, financial assistance or compassionate drug access, and drug infusion and injection services, and frequently serve as a bridge for patient access to novel drugs while awaiting public reimbursement [6].

Most specialty medicines launched in Canada have an associated PSP. With more than 400 PSPs currently in place [3], of which 175 support oncology drugs [4], Canada now has a well-established PSP infrastructure. This ecosystem represents a unique opportunity to fill evidence gaps through analyses of real-world data (RWD), thereby supporting better health outcomes and strengthening Canada’s healthcare system. To fully capitalize on this opportunity, a shared acknowledgement and understanding of both the benefits and limitations of PSP data is needed so that stakeholders can be confident when using the data to support healthcare decision making.

In this perspective article, we explore data collection through PSPs in Canada and the potential to generate acceptable real-world evidence (RWE) with the robustness required to inform health-system decisions about treatment, access, and reimbursement. To support this examination, we analyzed six PSP RWE studies to illustrate their data characteristics and the health outcomes collected using the PSP infrastructure. A framework for collecting real-world data within a PSP and a checklist to address issues of trust and bias in PSP data collection are also examined.

## The value and limitations of patient support programs as a source of real-world data

Capturing data that is accurately representative of Canada’s diverse patient populations [5, 7] is critical for real-world evidence generation that will inform healthcare decision making. Through PSPs, a large percentage of Canadian patients can often be captured within the datasets, typically at a pan-Canadian level. A survey performed in 2019 suggests that once a patient enters a PSP, there is >75% patient retention within the program after 2 years or longer [8], making them a suitable vehicle for collecting longitudinal data. Moreover, PSPs are familiar to patients and health professionals, and the basic infrastructure for data collection already exists within the PSP ecosystem.

Collecting data from PSPs is not a new concept, and recent years have seen an evolution in PSP data collection expertise and capabilities, with leading manufacturers investing in strengthening the PSP datasphere to include the tracking and analyzing of health outcomes data [9–11]. Gathering outcomes data from PSPs leverages the infrastructure of these programs.

A key feature of PSPs is their design flexibility: both patient services and data collection can be tailored to the clinical needs of a specific patient population. Manufacturers and PSP vendors can evaluate PSPs at the design phase, identify the data needed to address Canadian evidence gaps, and tailor their data collection plans accordingly. Types of data collected through PSPs may include, among others [11, 12]:

• Baseline patient demographics
• Disease characteristics
• Treatment patterns
• Clinical and quality-of-life outcomes
• Reasons for treatment discontinuation

However, PSP data does have limitations. Patient registries typically capture data from multiple drugs within a therapeutic area, which allows for comparative analyses from the same data set. PSPs, however, are tethered to a particular drug and typically do not facilitate data collection that would enable comparative analysis. Additionally, each PSP design is unique and collecting its own data set, which can lead to differences in the data generated across programs.

As an industry-generated data source, PSP data may be seen as having a built-in bias, and this perception may reduce stakeholders’ trust in the data. Indeed, Canadian health technology assessment (HTA) bodies and payers have identified bias and trust as key concerns when making healthcare decisions based on RWE, including PSP data [13]. However, with improved and more objective data collection methodologies to eliminate bias and a prospectively designed analysis plan as discussed below, future acceptance of PSP as a valid source of RWE could be a reality.

## How is patient support program data being used for real-world evidence generation today?

**FIGURE 1:**
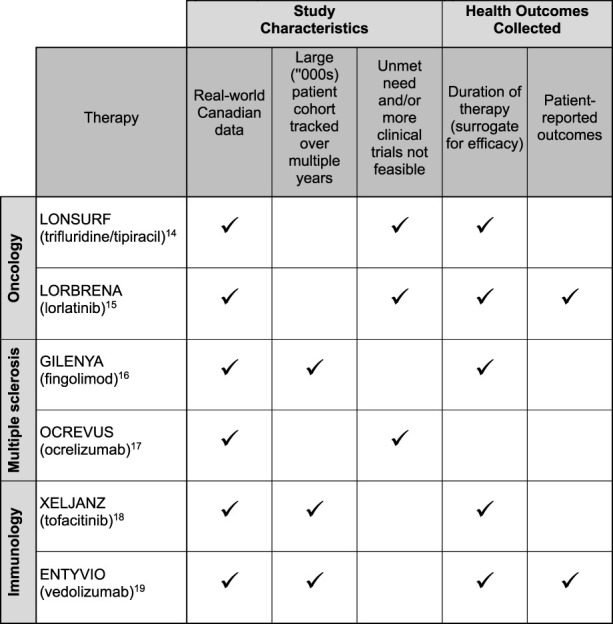
Study characteristics and health outcomes collected from six real-world evidence studies leveraging Canadian patient support program data.

To illustrate the current state of PSP data collection in Canada, Figure 1 lists six studies in which Canadian PSPs collected data [14–19]. Examples of types of data collected through the PSPs in these studies include patient characteristics, prior therapies, lab results, doses and dosing changes, reasons for discontinuation, and treatment outcomes. Duration of therapy, an outcome in five of the six studies, serves as a surrogate for efficacy. Patient-reported outcomes collected include the Harvey-Bradshaw Index, Partial Mayo Score, and EuroQol-5D (EQ5D). Half of these studies [14, 15, 17] filled an evidence gap where there was a high unmet patient need and/or an inability to conduct additional randomized controlled trials due to ethical concerns or limited patient populations.

A closer look at 3 of the studies [14, 17, 19] in Figure 1 illustrates the range of RWD that can be captured from PSPs:

• Case 1: *OCREVUS (ocrelizumab) for multiple sclerosis.* [17] The study assessed pregnancy outcomes in women with MS treated with ocrelizumab. Data was collected from the medication’s PSP and the manufacturer’s Global Safety Database. Combining these two data sources made it possible to study the safety of ocrelizumab before and during pregnancy in patients with MS. This study filled an important evidence gap as clinical studies may not be feasible for this patient subpopulation. Data collected from the PSP included age, type of MS, previous disease-modifying therapy, dates of ocrelizumab infusions, and duration of ocrelizumab therapy prior to conception. The safety database provided information on the indication for ocrelizumab therapy, estimated date of conception, number of previous live births, number of previous spontaneous abortions or pregnancy risk factors, contraceptive use, and decisions about getting pregnant and breastfeeding. Pregnancy outcomes data were extracted from the narratives of pregnancy cases reported in both data sets, with live birth, spontaneous abortion, ectopic pregnancy, and therapeutic/elective termination outcomes reported in the study.
• Case 2: *LONSURF (trifluridine/tipiracil) for metastatic stomach cancer.* [14] The study assessed the use of trifluridine/tipiracil (TFD/TPI) for the management of metastatic gastric and gastroesophageal junction cancers. TFD/TPI represents Canada’s first standard-of-care, third-line systemic therapy for these cancers. This study followed 123 patients on TFD/TPI over 2 years, with the PSP collecting baseline data on age, primary diagnosis, HER2 status, and prior therapies, as well as treatment data including therapy start and stop dates, doses, dose adjustments, and reasons for discontinuing treatment. The PSP also enabled the assessment of reimbursement status, including compassionate access to therapy, and a comparison of time to reimbursement between public and private drug plans.
• Case 3: *ENTYVIO (vedolizumab) for inflammatory bowel disease (IBD).* [19] The study looked at real-world outcomes and laboratory parameters in patients with IBD (including Crohn’s disease and ulcerative colitis) treated with vedolizumab. The study followed patients enrolled in the vedolizumab PSP between 2018 and 2020, compiling baseline, laboratory, and treatment data at defined timepoints. Patient characteristics collected in the study include age, sex, disease subtype, and prior exposure to a biologic therapy. The study also collected laboratory results, such as the trough concentration of post-induction vedolizumab at week 14, albumin at baseline, and fecal calprotectin and C-reactive protein at week 0 and 30. The Harvey-Bradshaw Index and Partial Mayo Scores were likewise recorded at week 0 and 30. Study investigators correlated these variables to remission, which they defined in one of three ways: C-reactive protein <5 mg/L, fecal calprotectin < 250 μg/g, or Partial Mayo Scores < 3 at week 30.

## What advances have been made in patient support program data collection processes? A recent observation

In recent years we have observed improvements in PSP data collection processes, notably in the areas of patient consent, data collection at enrolment, and incorporating data collection into program design.

**FIGURE 2:**
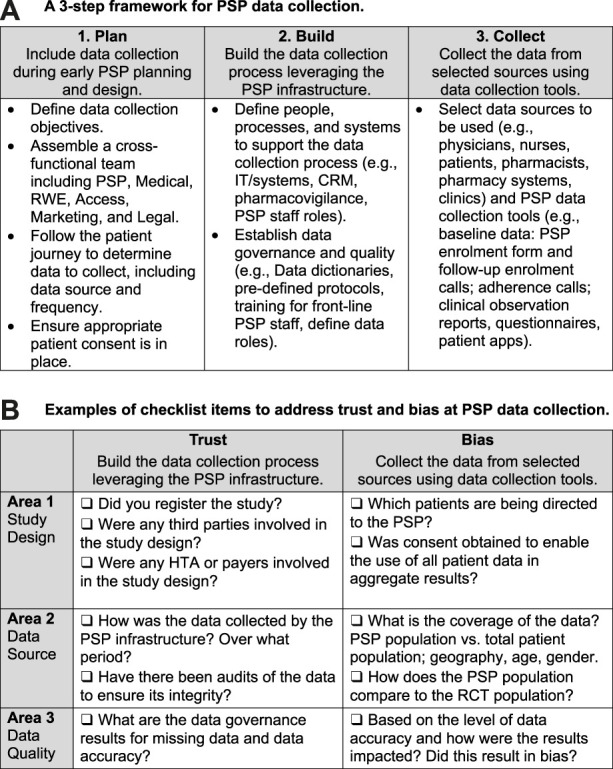
Tools for collecting real-world data within a patient support program. **(A)** A 3-step framework for PSP data collection. **(B)** Examples of checklist items to address trust and bias at PSP data collection.

Area 1: *Patient consent for data collection and use*. Appropriate informed and engaged patient consent must be obtained to enable PSP data collection and use, with the patient consent processes prioritizing plain-language and simplicity. A review of consent forms for PSPs from which data were published in peer-reviewed research found that consent statements are customized rather than using a “standard” format, and that one-stop consent processes are being used [11]. Slight differences in language can be traced to each organization’s legal requirements and internal editorial guidelines. All consent statements contain language with similar themes, specifying that the data will be “de-identified,” “anonymized,” and/or “aggregated” when used for analysis. The consent statements also provide examples of how the data might be used, including for research, publication, or commercial insights. Furthermore, some manufacturers and PSP service providers are working to involve patients in decisions around data collection, governance, and use [11].
Area 2: *Baseline data collection at enrolment.* The PSP enrolment form is used as the key vehicle for collecting baseline information, sometimes followed by telephone contact with the patient to confirm and supplement the enrolment information [11]. Data readily captured in the PSP enrolment form include patient demographics (e.g., age and gender), prior therapies, information about healthcare providers, lab test results including gene or biomarker status, disease and quality-of-life scores, clinical data, and payer information, among others. Manufacturers have identified several best practices for data collection via PSP enrolment forms. They agree on the importance of a simple and clear layout, divided into sections. A 2-page format, with one page for collecting information and the other for administrative requirements (e.g., consent and disclaimers) helps organize the information. Many programs use fillable PDF enrolment forms with tick boxes and drop-down menus, which take less time to complete, help standardize the datasets, and facilitate reliable data capture for analysis.
Area 3: *PSPs that incorporate data collection into their design at program setup.* Forward-thinking manufacturers and PSP vendors are further increasing data quality by building data collection into the design of their PSP. As shown in Figure 2A, integrating data collection into a PSP can be done via a three-step process: plan, build, and collect.

In the planning phase, the manufacturer defines the data collection objectives and assembles a cross-functional team to determine the data to collect, including the source (where to collect it), method (how to collect it), and frequency (when to collect it). For example, if the data source is a lab test, the team would determine whether to collect the data from the patient, doctor, and/or lab, and at what points in the patient journey to collect it. The cross-functional team may include representatives from the manufacturer’s medical affairs, marketing, market access, real-world evidence, and legal divisions, as well as personnel from the PSP vendor, supplemented by consultations with patients, physicians, and data and analytics experts.

In the building phase, team members determine how best to leverage the PSP infrastructure to collect and assess the needed data. They then establish data collection processes that align with touchpoints in the existing PSP infrastructure, such as scheduled patient interactions with coordinators, nurses, pharmacists, and infusion clinics. The building phase also includes the creation of controls to ensure the quality and integrity of the data. Chief among them are establishing pre-defined protocols, conducting regular monitoring through audits, and training front-line PSP staff on data entry. Quality assurance controls should be defined, including data-specific roles (e.g., data controller) and data quality metrics for the PSP vendors collecting the data.

At the data collection phase, the PSP gathers data from such sources as physicians, nurses, pharmacists, pharmacy systems, clinics, and patients. Along with enrolment forms and follow-up calls, PSP data collection tools may include post-infusion or post-injection reports, adherence calls, and patient questionnaires.

Some manufacturers have added quality assurance control checkpoints upon receipt of the data and regular review protocols that include data quality measures [11].

## Is real-world evidence from patient support programs acceptable for healthcare decision making today?

Some manufacturers have indicated that RWD from PSPs can help guide their strategies, and also report that physicians find real-world studies from PSP data helpful to inform them about Canadian patient populations [11]. Conversely, current PSP data has experienced limited acceptability as RWE to inform decisions for assessments and reimbursement—for example, by HTA bodies, the pan-Canadian Pharmaceutical Alliance, and payers. In principle, these stakeholders are open to considering PSP data, but they need a greater understanding of, and confidence in, the quality of the data in order to use it [20].

One concern about PSP data, common to all observational databases, is that it lacks the rigor of controlled clinical trial data [21]. Another is that an industry-funded data source may have a bias. Bias, in relation to data, can be defined as concerns about unfair prejudice in favor or against one parameter, person, or group when compared with another [22]. Selection bias ranks high among stakeholder concerns, along with missing data bias and publication bias. In real-world studies, selection bias may arise when the data is collected from a small number of centers or limited catchment areas [23]. Disparities between the study cohort and the target population for a treatment, for example, is another example of a data-related bias [22].

Bias, true or perceived, weakens decision makers’ trust in the data. Trust issues stem from concerns about the reliability of the data and the resulting evidence generated from it—for example, when there are suspicions of data dredging or cherry picking [22]. Additional concerns about trust include the integrity of the data and whether it can be audited and validated.

Given these challenges, it is critical to investigate how trust and bias in PSP data collection can be addressed. As previously noted, the research community has begun to address trust and bias issues by engaging independent academic researchers to oversee PSP design and implementation, supervise data collection and quality assurance, and analyze and validate PSP data. In some instances, researchers are linking PSP data to other datasets, such as Health Canada’s Special Access Program data [24] and global safety databases [17]. A few are using predictive analytics and other advanced research methods to arrive at key safety and efficacy outcomes—including overall survival [14].

Some countries have developed guidelines for RWE generation, and following such guidelines provides an extra level of mitigation against bias and trust concerns. For example, the UK’s 2022 NICE real-world evidence framework identifies “when real-world data can be used to reduce uncertainties and improve guidance” and describes best practices for planning, conducting and reporting RWE studies [22]. In May 2023, the Canadian Agency for Drugs and Technology in Health (CADTH) published a guidance document on the reporting of RWE that can be considered for regulatory and reimbursement decisions [25]. While such guidelines do not guarantee acceptable RWE data, they include tools, such as templates and questionnaires, that facilitate evaluation of the data and are likely to increase its acceptability. PSP data developers should use such guidelines to understand the criteria that make RWE acceptable, to counteract sources of bias, and to address gaps in the trustworthiness of a dataset.

Figure 2B is a checklist that can help PSP data developers address trust and bias issues by increasing transparency in RWE studies, with a focus on three areas: study design, data source, and data quality [11, 22]. Ideally, any PSP RWE study submission to reviewers should provide details corresponding to each of the checklist items.

## Discussion: next steps for patient support program data collection to enable high-quality, acceptable, real-world evidence

A good structure requires a strong foundation. Transposed to the realm of PSP data, this means that quality of RWE generation from PSPs must get stronger before healthcare decision makers can be expected to accept it. Only when bias and trust issues are addressed will decision makers have the confidence that PSP datasets reflect real-world scenarios.

To this end, PSP developers can set up their programs to collect clinical data from the start—an approach already adopted by some industry leaders, both in Canada and internationally. Dr. Aastha Dolley, Senior Director, Medical at Taiho Canada, on the experience of leveraging the PSP to collect data noted that “we are extremely selective in what we include in our PSP data collection plans, to ensure we get all the information we need to conduct robust research.” [26] In Switzerland, Novartis has “developed a company-wide guidance to collect baseline patient data and prospective follow-up information at product resupply” from their PSPs as data from these programs are “increasingly considered as a source of real-world data.” [12] Establishing data collection as a norm for PSPs will require collaboration from manufacturers, PSP providers, and data experts. Together, they can learn from previous efforts and generate the momentum needed to reach the threshold of acceptance.

Forward-thinking stakeholders are already conducting studies that use PSP data to gain insights into real-world clinical and quality-of-life outcomes [14, 23, 27]. Sharing and critiquing such studies enables researchers to identify and address data quality issues. Registering RWE studies in advance and systematically publishing study results, an approach recommended by the International Society for Pharmacoeconomics and Outcomes Research [28], can further increase trust and reduce bias.

Returning to the key area of examination of this article—looking at data collection within patient support programs in Canada and the implications for real-world evidence generation—at present, the data from each PSP must be considered on its own merits, given that not all PSPs are alike in their design and current capabilities. While PSP developers have a general idea of how to optimize the data they collect, internal legal meant to limit medico-legal risk might impede the process. In this case, specific criteria for data acceptability would help guide their efforts. Perhaps a pan-Canadian guidance document on PSP data collection and use for RWE generation, or an extension of CADTH’s RWE guidance document specific to PSP data, could help fill this gap.

Canadian PSP developers, patients and healthcare providers have expressed a consistent interest in unlocking the full value of PSP data and have an opportunity to build on previous efforts to carry PSP data to the highest level: acceptable for decision makers and transformative for patients.

## Data Availability

The original contributions presented in the study are included in the article/supplementary material, further inquiries can be directed to the corresponding author.

## Abbreviations

CADTH, Canadian Agency for Drugs and Technology in Health; EQ5D, EuroQol-5D, an instrument for measuring quality of life; HTA, Health technology assessment; MS, Multiple sclerosis; PSP, Patient support programs; RWD, Real-world data; RWE, Real-world evidence.
